# A Multilevel Logit Estimation on the Determinants of Utilization of Preventive Health Care and Healthy Lifestyle Practice in China

**DOI:** 10.1002/wmh3.160

**Published:** 2015-10-25

**Authors:** Lida Fan, Jianye Liu, Nazim N Habibov

**Affiliations:** School of Social Work at Lakehead University in Thunder BayOntario, Canada; Department of Sociology at Lakehead UniversityThunder Bay, Ontario, Canada; School of Social Work at the University of Windsor in WindsorOntario, Canada

**Keywords:** determinants, preventive health care, China

## Abstract

The purpose of this study is to provide policy implications by estimating the individual and community level determinants of preventive health-care utilization in China based upon data from the China Health and Nutrition Survey. Two different frameworks, a human capital model and a psychological-behavioral model, are tested using a multilevel logit estimation. The results demonstrate different patterns for medical and nonmedical preventive activities. There is a strong correlation between having medical insurance and utilizing preventive health services. For the usage of medical-related preventive health care (MP), age, gender, education, urban residence, and medical insurance are strong predictors. High income did not provide much of an increase in the usage level of MP, but the lack of income was a huge obstacle for low-income people to overcome. Community variation in number of facilities accounted for about one third of the total variation in the utilization of MP. The utilization of MP in China remains dependent upon the individual's social-economic conditions.

## Introduction

How do people deal with threats to their health? In the developed world, the answers to this long-standing question tend to be either behaviorally or cognitively oriented (Kirscht, 1983; Prentice-Dunn & Rogers, 1986). However, the utilization of preventive health care in developing countries can be substantially different from that in the developed world (Dupas, 2011). Preventive health-care activities are associated with socioeconomic conditions, and their use depends on the types of activities involved; for example, whether they require medical facilities or can be done by individuals. In this study, we will investigate the determinants of both medical preventive health care and healthy lifestyle practice in China, using a data set from a nationally representative survey.

“Prevention first” has been one of the major health-care policies in China since the 1950s. This policy was considered a success in improving the health status of the Chinese people in pre-reform China (Sidel & Sidel, 1982; World Health Organization, 1975). The merit of the pre-reform Chinese medical system was characterized by an emphasis on prevention: “low-cost, locally controlled health services and promoting accessible primary health care in rural areas” (Hillier & Shen, 1996, p. 258). The pre-reform era's health-care system was efficient in terms of the control of infectious diseases; despite pervasive low productivity and shortage of services in almost every area, Chinese people's health status improved throughout the 1980s and 1990s (Fang & Bloom, 2010; Shu & Yao, 1997). During the economic reform, the utilization of preventive health care became challenging for many people, especially people living in rural areas and people without medical insurance, because of decentralization and marketization. Financial decentralization limits the role of the government in providing public health programs. During the reform, the urban medical scheme has been financed mainly through municipal-level risk pooling for employees with basic medical insurance. The rural “New Cooperative Medical Scheme” (NCMS) initiated in 2003 focuses “almost exclusively on medical care and funds only a few preventive services” (Fang & Bloom, 2010, p. 32). This policy may lead individuals to a more curative care focus, since one of the fundamental changes lies in increasing out-of-pocket expense. Thus spending on preventive health care becomes more of a personal choice for future well-being, rather than a public policy arrangement as it was before the reform. Although the ongoing health-care reform initiated in 2009 aims to provide comprehensive universal health coverage for all citizens by 2020, individuals' incentives would remain an important element in the new system (Yip et al., 2012). In this way, it becomes more about the individual's investment in their own human capital, impacted both by the individual's understanding of the importance of preventive care, and their economic ability to carry the associated costs.

In recent years, an increasing literature has focused on various key factors affecting the utilization of health care in China (Gao, Raven, & Tang, 2007; Liu, Zhang, Lu, Kwon, & Quan, 2007; Qian, Pong, Yin, Nagarajan, & Meng, 2009; Wagstaff, Lindelow, & Hsiao, 2009; Zhang & Kanbur, 2005; Zhang, Wang, & Zhang, 2014). However, although early studies explored the preventive health-care system before and after the reform (Hillier, 1986; Hillier & Shen, 1996; Kaufmana & Jing, 2002), if we limit our searching area to the determinants or factors affecting the utilization of preventive health care, we still have the impression that we know little in this area. Among the very few studies on the determinants of preventive health care in China, van Dalen (2006) estimated a few socioeconomic variables, including insurance and wealth, using probit-estimation and instrumental variables (IV) based on the data of the China Health and Nutrition Survey 2004. Using probit-estimation he found that possessing health insurance increased the probability of using preventive health care by 3 percent, but this effect was not statistically significant per the results of the IV probit-estimation. He also found that being in the lowest 40 percent of population wealth would reduce the likelihood of using preventive health care by 1 percent. However, being in the top 20 percent wealth group did not have any effect when compared with the middle wealth group. Many issues in the areas of preventive health care in China remain unexplored.

One of the reasons that we should pay more attention to preventive health care is that it is an efficient way to improve the well-being of the population, compared with curative strategies. Since much disease is preventable, and “[p]reventable illness makes up approximately 70 percent of the burden of illness and the associated costs” of health care (Fries et al., 1993, p. 322), preventive care is an efficient way to reduce health risk and significantly improve health outcome (Harvey, 2001; Rose, 1992). Also, because preventive health care reduces the incidence rate of disease, it is considered to be far more cost-efficient than a curative strategy (Kaplan, 2000; Rose, 1992).

The paucity of literature in this area and the costs and benefits of preventive care motivate us to examine the determinants of utilization of preventive care in the context before the current health-care reform initiated in 2009 using the 2006 wave of the China Health and Nutrition Survey. We are going to estimate the determinants of preventive health care with Grossman's (1972) framework, in which individuals, as the producers of health, allocate their resources to produce health. The reason we test the determinants with Grossman's framework is that individual behaviors in the utilization of preventive health care are at least partly rational and predictable. As such, understanding the determinants of utilization of preventive health care can help policymakers formulate better strategies for improving health outcomes in the current health-care reform. Specifically, this paper attempts to answer the following research questions:

1. What are individuals' determinants for using medical preventive health care and non-medical healthy lifestyles in China?
2. Do community factors make a difference in determining the use of preventive health care?
3. What are the policy implications regarding the utilization of preventive health care?

The remainder of this paper is organized as follows: The next section describes the data set, analytic framework, and variables estimated. The third section discusses the results and provides research implications. Finally, the last section will summarize the findings.

### Theoretical Framework

Different theories and models have been used to explain the utilization of preventive health care, including human capital, psychological, and behavioral models. These models are based upon a variety of frameworks and assumptions.

The Grossman (1972) model recognizes that health is both a consumption good used for direct satisfaction and an investment good for higher productivity. Using this model, the utilization of preventive health care for individuals can be understood as an investment in human capital. Although individuals cannot “purchase” health from the market, they can prevent the occurrence and reduce the severity of certain diseases by diagnosing them in the latent stage through the use of preventive health care. That is, the return on investment with preventive health care is the improved health status through prevention and reduction in the risks of severe illness. Similar to an investment in physical capital, an individual makes a decision to undergo preventive health care based on vectors of their individual characteristics and social-economic conditions (Glanz, Rimer, & Viswanath, 2008). The Grossman model performs well when the preventive care activities are more medically focused in nature (Cropper, 1981; Kenkel, 1991, 1994; Wagstaff, 1986).

However, the utilization of preventive health care is not simply the “purchase of health.” Preventive health care differs from other commodities. First, uncertainty is intrinsic to health care, both in patient outcomes and in financial concerns. This nature makes health care different from other commodities (Cropper, 1977). Uncertainty always exists and you continue to remain unsure of whether an investment in health will yield a positive result. Second, individuals may not have the knowledge and necessary information in order to estimate the value and possible outcomes of heath care (McGuire, Henderson, & Mooney, 1988). Third, some preventive activities are based on a choice of lifestyle rather than a rational choice of investment.

The above discussion implies that the characteristics of different types of preventive health care are different. Preventive health care covers a wide range of activities, “undertaken by a person believing himself to be healthy, for the purpose of preventing disease or detecting it in an asymptomatic state” (Kasl & Cobb, 1966, p. 246). These activities can be classified in different ways (Gordon, 1987; National Institute on Drug Abuse, 1997). For the purposes of this study, we use a classification of the dichotomy of medical-related preventive health care (MP) and nonmedical healthy lifestyle (Lifestyle). MP includes treatments conducted in hospitals for the purpose of early detection and prevention of disease, including regular blood tests, scans, and immunizations. The utilization of Lifestyle reflects an individual's behavior-based activities, which reduce the probability of illness. These behaviors include a healthy diet, physical activity, and not smoking, and have been studied within the frameworks of psychology (Kirscht, 1983; Prentice-Dunn & Rogers, 1986), and classified as alternative health care in medical studies (Kelner & Wellman, 1997).

Since health care includes a wide range of activities, the same factors may have different effects on the utilization of medical and nonmedical healthy lifestyle. In this paper, we will estimate the effects of the same set of determinants on MP and Lifestyle separately. The research strategy of this study is to compare the performance of different theoretical frameworks on the utilization of preventive health care activities. The explanatory power of the Grossman model and that of the behavioral and lifestyle frameworks on MP and Lifestyle can be compared using the results of the estimation. Therefore, the complexity of preventive health-care activities can be analyzed in both rational and irrational frameworks.

## Data and Methods

### Data

The data sets used in this study are from the Adult and Community Files of the China Health and Nutrition Survey (CHNS) 2006. This publicly available survey, overseen by the Health Sciences Institutional Review Board at the University of North Carolina at Chapel Hill, and primarily funded by the National Institutes of Health (United States), is used to evaluate the effects of economic reforms, as well as ongoing government programs addressing public health and nutrition issues. This survey was conducted by the Carolina Population Center (CPC) at the University of North Carolina beginning in 1989. Using a multistage, random cluster sampling design, the data were collected from 4,446 households, with 11,742 individuals, in nine provinces of China, namely Guangxi, Guizhou, Heilongjiang, Henan, Hubei, Hunan, Jiangsu, Liaoning, and Shandong.

The advantages of using the Adult and Community files of the CHNS should be emphasized. The CHNS is a major survey done in China, containing a significant amount of information on demographics, health and health care, and socioeconomic indicators of individuals and communities. The Adult File contains a wide range of information about adults (aged 18 and older), including age, gender, education, urban-rural status, occupational activities, individual eating patterns, nutritional knowledge, daily health-related activities, health status, reproductive health, utilization of health care, medical insurance, and individual and household income. The Community File contains information regarding the socioeconomic situation of each community (such as educational and income profiles), and the medical conditions of the community (such as the number of medical facilities, the number of beds, and the number of doctors serving at these facilities) though there is no community weight variable that can be used to reflect weighted summary statistics based on the communities of the whole county. A community (village or neighborhood) is a government-designated administrative district, not a natural population cluster. There are 218 communities in the 2006 CNHS. Combining the Adult and Community Files provides a rich source of information for an evaluation of public-health policies. It satisfies the purpose of this study for understanding the social, demographic, and community determinants affecting MP utilization and Lifestyle practice.

### The Model

An individual's decision to use preventive health care can be studied as a choice between using preventive care services or engaging in other competing activities, based upon vectors of the individual's demographics, social-economic characteristics, and his/her socioeconomic environment.

To account for the community-level clustering in the CHNS data, a multilevel logit model is used. In constructing this model we assume that individuals will try to maximize their return on investment through their decision-making process. Then, following Rabe-Hesketh and Skrondal (2008, p. 247), the probability of an individual *i*, living in community *j*, using preventive health care, given his/her demographic characteristics, social-economic status, and community profile is described as follows:


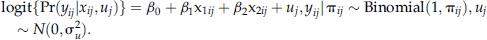


where *y_ij_* is binomially distributed with π_ij_ being the probability that the *i*th individual of the *j*th community utilized preventive health care; *x*_1_ is a vector of the individual's demographic characteristics, such as age and gender; and *x*_2_ is a vector of community variables, such as number of facilities in the community. The *u_j_* is the random error at the community level, assumed to be normally distributed, with a mean of 0 and variance of σ_*u*_^2^ (after taking the community-level covariates into account). This allows us to calculate the portion of the variation at the community level: ρ= σ_*u*_^2^/(σ_*u*_^2^ +σ_*k*_^2^), where σ_*k*_^2^ denotes the variance at the individual level, and ρ is known as the inter-class correlation. A higher value of ρ indicates a higher proportion of the total variance in utilization of preventive health care, derived from community level differences. This multilevel model permits the estimation of two groups of parameters: fixed and random effects. Fixed effects represent the relationship between the observed individual explanatory variables and the outcome variable (the utilization of preventive health care). In contrast, random effects do not come from the observed individual variables, but from the variations among communities, measured in terms of the value of ρ.

The estimation is conducted using the xtlogit command of STATA 10 software package (Rabe-Hesketh & Skrondal, 2008, p. 248). Four models are estimated. Model 1 is an empty model, measuring only the community-level variance, using community ID without any independent variables. Model 2 includes community variables only. Model 3 includes individual variables only. Finally, Model 4 incorporates both individual and community variables. These models produce consistent estimations of fixed and random effects. To conceptualize the determinants of the utilization of preventive health care in China, the remaining part of this section will discuss the specific measurements of the variables used in these models.

### Dependent Variables

#### Utilization of Medical Preventive Health Care (MP)

This is a binomial variable, defined by whether the respondent used any MP services within four weeks preceding the interview date of the CHNS. This includes health examinations, eye examinations, blood tests, blood-pressure screening, tumor screening, prenatal and postnatal examinations, and any other type of preventative examinations. The value is 1 if the respondent used one of these preventive services, otherwise the value is 0. The nature of these services is different from that of curative health care and from a Lifestyle focus. First, the individual's perception of MP is different from that of curative care, since MP is not as urgent as curative care, while people use curative care when they are sick. On the other hand, the use of preventive care can be delayed, and may yield to other competing consumptions and investments, given the constraints of money, time, and other resources. Second, MP differs from Lifestyle, because the former has to be done in a hospital and/or requires the use of medical equipment.

#### Practice of Nonmedical Healthy Lifestyle (Lifestyle)

The term “nonmedical healthy lifestyle” covers a wide range of practices dealing with illness and other forms of health care, often known as alternative health care, including physical manipulation, acupuncture and traditional Chinese medicine and naturopathy, vitamin supplements, and healthy diet (Kelner & Wellman, 1997). Given the availability of the information of the survey data and the preventive focus of this study, we use a narrow definition. This binomial variable is defined as whether the respondent performed certain health-related activities or had a healthy lifestyle. A value of 1 is applied if the person was a nonsmoker, who had not consumed alcohol in the past year, maintained healthy food choices, and performed regular physical activity. Otherwise a value of 0 is applied. Here, healthy food choice is an aggregated index calculated from four Likert-scale questions within the survey: fp = score of fruits preference + score of vegetable preference − score of fast food preference − score of salty snack food preference. A healthy food preference is indicative of a fp value greater than 0. Physical activities included one of the following: walking; Tai Chi practice; or participation in sports such as ping-pong, badminton, tennis, soccer, etc. This variable is labeled nonmedical healthy lifestyle, since all of the included activities are nonmedical in nature, and are related to personal lifestyle choices. These nonmedical behavior-related choices contribute to a large proportion of the population's health status (Mokdad, Marks, Stroup, & Gerberding, 2004).

We predict that there will be a significant difference between the results based on the same set of determinants when comparing the two dependent variables: MP and Lifestyle.

### Independent Variables

We use of the same set independent variables to estimate both medical and nonmedical dependent variables for comparative purposes. It may be obvious that individuals need stronger financial status to utilize medical preventive health care than to practice nonmedical healthy lifestyles. For example, people do not necessarily need to be rich or have medical insurance in order to practice healthy lifestyles. However, it is still useful to know how these independent variables affect the utilization of preventive heath care empirically.

#### Age

Age is one of the most important characteristics in predicting the utilization of health care in Andersen's (1995) behavior model. This is due to the predisposition of elderly individuals to degenerative diseases, resulting from “biological imperatives.” However, the relationship between age and utilization of preventive care, as is a focus of this study, is complicated in terms of investment in human capital. Young people tend to invest more in human capital (e.g., education and health) versus the elderly, simply because they have a longer term in which to earn a return (Cropper, 1977). This propensity however, may be lowered as a result of two other processes. First, young people have several competing investments in human capital, aside from health (e.g., education and physical capital). Quite often young people have to choose to invest in different dimensions of human capital at the expense of health care. An example of this is evident in that a high rate of return on investment in education may limit or prevent an investment in health. Second, younger people in general have better health status than the elderly and thus they are less concerned about their health, and therefore tend to invest in it less. Although the combined results of these competing factors can be complex, we hypothesize that age is positively associated with the utilization of both MP and Lifestyle.

#### Female

There has been a longstanding belief that women in the developed world are more likely than men to use MP (Kirscht, 1983; Lairson & Swint, 1978). However, the gender gap in employment and education can be an important factor affecting the utilization of health care in developing countries. Women traditionally remain out of the formal labor market and are more likely to be involved in unpaid housework and family responsibilities. For this reason, women are less likely to obtain higher education, and thus have decreased opportunities for workplace medical insurance and lower levels of income to use for health-care expenses in developing countries (Fan & Habibov, 2009). For this dummy variable, male is given the value 0 and female is 1. Given the fact that a significant gender gap still exists in China, we are not sure about impact of being a female on the use of MP. However, we can predict that being a female increases the likelihood of practicing Lifestyle.

#### High Education

Education is the most important “environmental variable” affecting the production of both health and home goods, per the Grossman (1972) model. People with higher levels of education have more knowledge about the importance of utilizing preventive health care and will possess more resources with which to invest in health. For these reasons, people with a higher level of education are more likely to use both MP and Lifestyle than those with lower levels of education.

This dummy variable takes the value 1 if the respondent received higher than lower-middle school education, and takes the value 0 if otherwise.

#### Urban Residence (Urban=1/Rural=0)

The huge urban-rural gap in China, also known as the dual economy, was a result of the well-known Chinese Household Registration System that occurred prior to the reform. It remains in place today, not only in terms of income but also in relation to social services, including medical services. Urban Residence is a dummy variable that takes the value of 1 for an individual living in an urban area and 0 for an individual living in a rural area. Place of residence becomes especially relevant to the use of preventive health care services in China, given the huge rural-urban disparities in health care education, accessibility of social welfare, and the availability of health-care facilities.

#### Medical Insurance (Yes=1/No=0)

Insurance can be an enabling factor in the utilization of health care, according to the Andersen Behavior Model (1995). However, as discussed earlier, studies on the role of insurance in the utilization of health care in China are mixed (Akin, Dow, & Lance, 2004; Liu et al., 2007; Wagstaff et al., 2009; Wong, Tang & Lo, 2007; Zhu, Zhu, & Liu 2008;). We predict that people having health insurance are more likely to use MP, but not necessarily more likely to use Lifestyle.

#### Low Household Income PC

This dummy variable takes the value 1 if the individual's per capita household income is in the lowest quartile (the lowest 25 percent of the households in the sample). In China, individuals in the lowest quartile have limited buying power with which to maintain their daily activities. In our sample, the cut-off annual per capita household income is 2050 *yuan* (approximately $255 U.S. in 2006). People in this quartile have difficulties meeting their basic need for food. If they do not have insurance coverage, preventive health care is considered a luxury. For them, preventive health care may not be their priority and the service charges for a basic general physical examination may be beyond their monthly living spending. We predict that being in the lowest quartile of household per capita income limits the affordability of both MP and Lifestyle.

#### High Household Income PC

This is a dummy variable defined as 1 if the per capita household income is in the highest quartile. High household income increases the ability to financially manage the costs of preventive health care. We predict that being in the top quartile increases the opportunities for an individual to use preventive health care.

#### Distance to the Nearest Facility

Utilization of preventive health care increases with this supply factor. Having to travel to a health-care facility that is far from an individual's residence can seriously discourage the utilization of health care (Khan, Hotchkiss, Berruti, & Hutchinson, 2006). For the purposes of this study, we use Distance to the Nearest Facility as a supply factor, or in other words, the accessibility of the health-care service. It is expected that with an increase in distance to the nearest facility, the accessibility of MP would decrease. However, this should not affect Lifestyle.

#### Number of Facilities in the Community

This variable is defined as its name suggests. It is a measurement of the availability of health-care facilities at the community level. The study on the role of the community level supply factor (health-care facilities) is well documented for developing countries (Gwatkin et al., 2007). The lack of health-care facilities in their community forces people to travel a long distance in order to seek health care. We expect that the number of facilities available to the community is positively associated with MP. However, Lifestyle should not be significantly affected by it.

#### Per Capita Income of the Community

This is an indicator of the general level of the social-economic condition of the community. The level of per capita income at the community level is associated with many other factors such as: medical infrastructure, transportation, and the community's general social development level, including health education and knowledge of preventive health care. This variable is defined as per capita income of the community in 1000 RMB *yuan*. We predict that this variable is positively associated with both MP and Lifestyle. For more details about the variables, please see Table 1.

**Table 1:** Definitions and Descriptive Statistics of Independent and Dependent Variables

Variables	Definitions	Obs	Min	Max	Mean	SD
*Dependent variables*						
Utilization of medical preventive health care (MP)	Dummy variable = 1 if the individual received preventive health service, such as health examination, blood test, blood pressure screening, tumor screening in the last four weeks	9773	0	1	0.0336	0.1801
Practice of non-medical healthy lifestyle	Dummy variable = 1 if the respondent was a non-smoker, did not consume alcohol in the past year, maintained healthy food choices and performed regular physical activity	9773	0	1	0.1650	0.3712
*Independent variables*						
Age	Continuous variable of age (years) of the individual	9773	17	99	48.82	15.34
Female	Dummy variable = 1 if the individual was female	9773	0	1	0.5244	0.4943
High education	Dummy variable = 1 if the individual had lower-middle school education	9773	0	1	0.2450	0.4301
Urban residence	Dummy variable = 1 if the individual lived in an urban area	9773	0	1	0.3433	0.4748
Medical insurance	Dummy variable = 1 if the individual had medical insurance	9773	0	1	0.4936	0.5000
Low household income pc	Dummy variable = 1 if the individual's per capita household is in the lowest quartile	9773	0	1	0.2508	0.4345
High household income pc	Dummy variable = 1 if the individual's per capita household is in the highest quartile	9773	0	1	0.2500	0.4332
Distance to the nearest facility	Continuous variable that indicates distance in kilometers from home to nearest medical facility	9773	0	6.5	0.3294	0.8327
Number of facilities	Continuous variable, number of facilities in the community	9773	1	10	2.379	1.286
Per capita income of the community	Continuous variable, per capita income (in 1,000 RMB *yuan*) of the community	9773	400	14400	3857	2047

*Note*: Before recoding into related variables listed in the table, missing values of education, per capita income of the household, distance to the nearest facility, per capita income of the community were replaced with the mean.

*Source*: Authors' estimations based on CHNS (2006).

## Results

### Utilization of MP

The estimation results from the multilevel regression for MP are reported in Table 2. The intra-class correlation (ρ) reported in model 1 (the empty model) shows that a significant proportion (36.93 percent), of the total variation in using MP comes from the community level with a statistical significance at the 0.01 level. This indicates that the variations among communities using MP may be the result of different resources such as financial ability, level of development, and the infrastructure of medical facilities.

**Table 2:** Multilevel Logistic Regression Estimates: Fixed Effect (Odds Ratios) and Random Effects of Utilization of Medical Preventive Health Care (MP)

Variables	Model 1	Model 2	Model 3	Model 4
*Fixed effect*				
Individual variables (Level 1)				
Age			1.0157*** (0.0043)	1.0156*** (0.0043)
Female			1.6104*** (0.1968)	1.6091*** (0.1966)
High education			1.5575*** (0.2404)	1.5516*** (0.2392)
Urban residence			2.0237*** (0.4961)	1.7310** (0.4820)
Medical insurance			1.7768*** (0.2815)	1.7902*** (0.2858)
Low household income pc			0.7104* (0.1254)	0.7251* (0.1283)
High household income pc			1.1472 (0.1772)	1.1289 (0.1747)
Distance to the nearest facility			0.9565 (0.1470)	1.0116 (0.1531)
Level 2 (community variables)				
Number of facilities		1.1892** (0.1030)		1.2112** (0.1015)
Community per capita income		1.0002*** (0.0001)		1.0001 (0.0001)
*Random effect*				
Intra-class correlation, ρ	0.3693***(0.0438)	0.3432***(0.0428)	0.3255*** (0.0427)	0.3172*** (0.0423)
Log-likelihood	−1311.08	−1308.18	−1275.93	−1272.64
Number of observations	9773	9773	9773	9773

**Notes**: Odd ratios are reported, standard errors are in parentheses.

*p < 0.10,

**p < 0.05,

***p < 0.01.

Results from Model 2 partly confirm the variations noted at the community level. The estimation results indicate that the Number of Facilities in the Community is positively associated with MP, and this relationship is statistically significant at the 0.05 level. The odds ratio for this variable is 1.1892, meaning that for each increase of one facility, the probability of using MP increases by 18.92 percent. However, Community per Capita Income had almost no effect on MP. The combined results from Model 2 suggest that community level variation is mainly caused by supply factors rather than economic development level. Therefore, communities with similar economic conditions could benefit through the provision of resources for MP services. Next we will discuss the individual factors reported in Model 3.

First, the age of the individual is positively related to MP at the 0.01 significance level. The odds ratio of Age indicates that for every year an individual's age increases, the likelihood of using MP increases by 1.57 percent. This is in line with the Andersen (1995) model, in which older people tend to be less healthy than younger people, and they tend to use more medical preventive services.

Second, the variable Female has a strong positive relationship with MP (with a significance level of 0.01). The odds ratio indicates that the probability of a female using MP is about 61 percent higher than it is for a male. This is in line with previous studies focusing on the background of developed countries (Kirscht, 1983).

Third, the result for High Education shows that the possession of high education moderately increases the probability of using medical preventive health care by about 55.75 percent. This relation is statistically significant at the 0.01 level. Education is an important variable in the Grossman model, and our study confirms that people with higher education are more likely to use preventive health care.

Fourth, living in an urban area radically increases the prospect of using medical preventive services, by about 102 percent. The high odds ratio for this variable reflects the huge urban-rural gap that exists on all dimensions of the social-economic conditions in contemporary China inherited from the household registration system. This gap is not expected to close for a considerably long period of time due to a few fundamental reasons (Chan & Buckingham, 2008).

Fifth, the results of our model estimation indicate that having medical insurance is significantly associated with the utilization of preventive health care at the 0.01 level. Having medical insurance improves the individual's likelihood of using MP by as much as 77.68 percent compared to those having no medical insurance.

Sixth, the estimation of the two variables of household income, Low Household Income pc and High Household Income pc, demonstrates some interesting results. People in the lowest quartile, well below the World Bank absolute poverty line, only had a 71.04 percent probability of using preventive health care compared with those in the middle two quartiles, with a statistical significance level of 0.1. In other words, if we can lift a person from the lowest quartile, up to the middle two quartile levels (the reference group) of earnings of the remaining population, his or her likelihood of using medical preventive health services would increase by 40.76 percent ((100–71.04)/71.04). However, the relationship between being in the highest quartile and the utilization of MP is not statistically significant. The results from these two variables demonstrate that having a high income level does not necessarily increase the utilization of preventive health care as much as would be evident through raising the income level of the individual person from the lowest quartile.

Finally, the Distance to the Nearest Facility variable is not statistically significant.

From a comparison of the results of all four models in Table 2, we can see a moderate change in the value of ρ from the empty model to Model 4. A comparison of Model 3 and Model 4 (the full model) generally shows consistent and similar results for the variables estimated, as well as for the random effects. This indicates that multi-collinearity is limited.

### Utilization of Lifestyle

The estimation results for Lifestyle presented in Table 3 show that the intra-class correlation (ρ) is only about 20 percent (Model 1), compared with about 37 percent for MP. This indicates a moderate intercommunity variation among the total variation of Lifestyle. Results from Model 3 also show that the odds ratios for the two community variables are close to 1, and are statistically insignificant. These results demonstrate that the community level variations have less impact on the utilization of Lifestyle.

**Table 3:** Multilevel Logistic Regression Estimates: Fixed Effect (Odds Ratios) and Random Effects of Practice of Nonmedical Healthy Lifestyle (Lifestyle)

Variables	Model 1	Model 2	Model 3	Model 4
*Fixed effect*				
Individual variables (level 1)				
Age			0.9966 (0.0021)	0.9966 (0.0021)
Female			4.6333*** (0.3154)	4.6301*** (0.3151)
High education			1.2162** (0.0980)	1.2160** (0.0980)
Urban residence			2.2107*** (0.2987)	1.9944*** (0.3049)
Medical insurance			1.1185 (0.0866)	1.1091 (0.0863)
Low household income pc			0.7966*** (0.0670)	0.8014*** (0.0675)
High household income pc			1.1798** (0.0932)	1.1720** (0.0927)
Distance to the nearest facility			1.1965** (0.0907)	1.2104**(0.0920)
Level 2 (community variables)				
Number of facilities		0.9877 (0.0258)		1.0346 (0.0516)
Community per capita income		0.9999 (0.0028)		1.0001 (0.0003)
*Random effect*				
Intra-class correlation, ρ	0.2006*** (0.0227)	0.1673*** (0.0210)	0.1633*** (0.0208)	0.1618*** (0.0207)
Log-likelihood	−4144.21	−4133.80	−3814.65	−3816.23
Number of observations	9733	9733	9733	9773

**Notes**: Odd ratios are reported, standard errors are in parentheses.

*p < 0.10,

**p < 0.05,

***p < 0.01.

Six individual variables, Female, High Education, Urban Residence, two household income variables, and Distance to the Nearest Facility, are statistically significant, as reported in Model 3 of Table 3. Two individual variables, Age and Medical Insurance, are not related to the use of Lifestyle statistically, as predicated.

First, among the significant variables, Female has a very strong positive relationship with the practice of Lifestyle at the 0.01 level. The odds ratio reveals that the probability of a female using these practices is 3.63 times higher than that of a male. This result is expected, since the dependent variable Lifestyle contains a measure of whether the individual smokes or drinks alcohol, as two of the four measured activities, and smokers and alcohol users are predominantly male. For example, male and female smokers present a prevalence ratio of 22 to 1 in China in 2010 (Li, Hsia, & Yang, 2011). Drinking alcohol is deeply rooted in a male-dominated tradition, with the ability to drink being considered a feature of being a man.

Second, the results indicate that having high education increases the probability of practicing Lifestyle by 21.62 percent. Education is widely believed to be an important factor influencing preventive health care, as is demonstrated in both the Grossman and behavior models. People with higher education not only have more knowledge on the importance of using preventive health care, but they also tend to choose healthier lifestyles. The possession of high education is not only a means to a good job, but also means more knowledge of the consequences surrounding particular lifestyles, including chosen activities. Thus, it provides a way of changing perceptions and encouraging choice of a healthy style of living.

Third, living in an urban area increases the odds of practicing Lifestyle services by about 1.2 times more than living in rural area, similar to that for MP. This means that although the practice of Lifestyle is not dependent upon urban medical facilities, urban environments are still important for practicing Lifestyle.

Fourth, the estimation of the two variables of household income, Low Household Income pc and High Household Income pc, demonstrates a similar pattern as that for the MP estimation. People in the lowest quartile had a 79.66 percent probability of using preventive health care, with statistical significance at the 0.01 level. It is understandable that people in economic difficulties also have difficulties in maintaining a healthy life style. The relationship between being in the highest quartile and the utilization of MP is statistically significant at the 0.05 level. The results from these two variables demonstrate that household income does matter in Lifestyle practice.

Fifth, Distance to the Nearest Facility has about a 20 percent effect on performing Lifestyle, with a statistical significance at the 0.05 level. Since practicing healthy lifestyle does not have a direct relationship with the distance to the facility, the mechanism for this relationship is empirically unclear.

Our results show that the practice of Lifestyle is impacted by the following factors: the individual's gender, education, where they reside, and household income. It is, more importantly, about a person's choice, rather than supply factors.

From a comparison of the two estimations reported in Tables 2 and 3, we can see that the same set of independent variables have differing impacts upon the two dependent variables. For MP, Age, Female, High Education, Urban, Medical Insurance, Low Household Income pc (though only at the .1 level), and Number of Facilities are significant variables. For Lifestyle, important factors determining lifestyle and consumption preferences also include being female, high education, living in urban areas, and household income; but unlike in the case of MP, distance to nearest facility also proves to be a significant factor, whereas age and medical insurance do not. Thus, in both cases some factors beyond the individual's control, such as economic conditions and availability of facilities, are important.

## Conclusions and Policy Implications

Through an analysis of the China Health and Nutrition Survey 2006, this study estimates the demographic, economic, social, and community determinants of the utilization of medical preventive health care (MP) and nonmedical healthy lifestyle (Lifestyle) in China. The determinants are constructed in an effort to test alternative economic and behavior frameworks. The major findings, as well as their policy implications, from our multilevel logit estimation, are summarized below.

First, for the usage of MP, age, gender, education, urban residence and medical insurance are strong predictors. Among these predictors, urban residence has a substantial impact on the likelihood of using medical preventive services. Although the urban-rural gap has been longstanding and will remain a hallmark of the Chinese dual-economic system for a considerable length of time, it is still vital that new initiatives in rural health improve upon these current conditions (Eggleston, Li, Meng, Lindelow, & Wagstaff, 2008; Xu, Zhang, & Zhu 2008). The results indicate that there is a strong correlation between having medical insurance and utilizing preventive health services. This implies that there is a great potential for improving the level of utilization of preventive health service through increasing medical insurance coverage, since more than 50 percent of the participants in the sample did not have any type of medical insurance coverage.

Second, the model estimation for the two household income variables demonstrates that having a high income did not improve the utilization level of MP as much as was evident in lifting the individual up from the lowest income quartile. In other words, high income did not provide much of an increase in the usage level of MP, but the lack of income was a huge obstacle for low-income people to overcome. In this sense, poverty reduction is an efficient measure that could significantly improve the utilization of MP and eventually improve health status for the population.

Third, the multilevel model estimation shows that community variation accounted for about one third of the total variation in the utilization of MP. The number of facilities, rather than the community per capita income, is a good community predictor. Thus under the same economic conditions, improvements to the infrastructure of medical facilities can make a significant difference.

Fourth, in considering the practice of nonmedical healthy lifestyle, gender, education, urban residence and household income are important predictors. Although these lifestyle-related activities are “nonmedical”, they are crucial to the health status of the general population. Cancer, heart diseases, and cerebrovascular disease—the top three causes of death in China in 2000—are all related to diet preferences, smoking, alcohol consumption, and a lack of physical activities. Lung cancer is the number one cause of death in China and smoking is a principal risk factor in lung cancer. Although the public has an increased awareness of the dangers of smoking, reducing smoking is a tough task. China is the largest tobacco producer and consumer in the world and this current pattern of economic development for a higher level of GDP could remain. The Chinese state-owned tobacco companies produce over 1.7 trillion cigarettes annually, which contributed 7.4 percent to the total government revenue in 2003 (Hu et al., 2005). Unless the government yields to health concerns, by changing the current GDP-oriented policy, there will not be a big move in reducing smoking behavior.

Fifth, the results of this study indicate that the Grossman framework performs very well for the use of MP. However, a behavior-based framework provides a more reasonable explanation for the practice of Lifestyle. The results of this study support the assertion that preventive health actions appear to represent several independent domains (Kirscht, 1983). Since social-economic factors have differing impacts on preventive health care activities, classifying these activities and choosing proper research frameworks are important in preventive health-care research. Further studies may consider classifications and theoretical frameworks other than the MP and Lifestyle dichotomy used in this study. It is essential to do so, since this would allow health-care policymakers to apply different strategies, targeting specific preventive activities, in order to improve the health status of their population.

Finally, the previously discussed results of our study indicate that the utilization of MP in China remains dependent upon the individual's social-economic conditions. However, MP should not simply rely upon the individual's ability and effort, since some infectious diseases could eventually become a public issue if no effective preventive care plan is in place. For example, currently there are about 120 million individuals infected with the hepatitis B virus (HBV) in China. This accounts for about 9 percent of the Chinese population and one-third of the total number of the HBV infections in the world (Custer et al., 2004; Liu & Fan, 2007). High-quality HBV vaccines are still too costly for low-income Chinese people. If the government were to provide this vaccination to all citizens, especially those without health insurance, a significant improvement could be achieved. Although the results of this study clearly indicate that the utilization of Lifestyle is partly a matter of an individual's personal characteristics such as education and gender, the government could still bring about significant change. MP and Lifestyle are different and not wholly substitutable for each other, thus they do not compete for the same resources of the government. For improvement of Lifestyle, the government could refrain from seeking profits from the tobacco and alcohol industry, and set new regulations for cigarette and liquor production and consumption, while smoking in public could be prohibited. In conclusion, all of these suggestions are based upon the assumption that the government would forego GDP growth as their sole objective.

One obvious limitation of this study is that the available information on medical preventive health care only accounts for limited items of preventive health care within four weeks preceding the interview date of the CHNS, which does not capture the comprehensive nature of preventive health care. In addition, it should be noted that the results of this study reflect the situation of health care before the health-care reform beginning in 2009. This reform aims to provide nearly universal health-care coverage by 2020. With the progress of the reform, more people will have medical insurance, and their health-related behaviors will change. The results of this study might be useful for new policy arrangements in providing information on behaviors in the case that most rural people did not have medical insurance. Preventive health care could be a more important component in the new health-care system if the government takes more responsibilities in the reformed system. Finally, it would be interesting to make comparisons on the changes of health-care behaviors during the reform in further study. Those comparisons would provide useful information for improving the efficiency of the new medical system.
